# Calling the amino acid sequence of a protein/peptide from the nanospectrum produced by a sub-nanometer diameter pore

**DOI:** 10.1038/s41598-022-22305-x

**Published:** 2022-10-25

**Authors:** Xiaowen Liu, Zhuxin Dong, Gregory Timp

**Affiliations:** 1 Tulane Center for Biomedical Informatics and Genomics, Tulane University, New Orleans, LA 70112 USA; 2 Division of Biomedical Informatics and Genomics, Deming Department of Medicine, Tulane University, New Orleans, LA 70112 USA; 3 Departments of Electrical Engineering and Biological Sciences, University of Notre Dame, Notre Dame, IN 46556 USA

**Keywords:** Biotechnology, Computational biology and bioinformatics

## Abstract

The blockade current that develops when a protein translocates across a thin membrane through a sub-nanometer diameter pore informs with extreme sensitivity on the sequence of amino acids that constitute the protein. The current blockade signals measured during the translocation are called a nanospectrum of the protein. Whereas mass spectrometry (MS) is still the dominant technology for protein identification, it suffers limitations. In proteome-wide studies, MS identifies proteins by database search but often fails to provide high protein sequence coverage. It is also not very sensitive requiring about a femtomole for protein identification. Compared with MS, a sub-nanometer diameter pore (i.e. a sub-nanopore) directly reads the amino acids constituting a single protein molecule, but efficient computational tools are still required for processing and interpreting nanospectra. Here, we delineate computational methods for processing sub-nanopore nanospectra and predicting theoretical nanospectra from protein sequences, which are essential for protein identification.

## Introduction

Sequencing proteins by measuring the blockade current through a sub-nanometer diameter pore (sub-nanopore) is a potentially disruptive technology^1–3^. So far, this technology has been successfully employed to analyze histones and other proteins^4,5^. A sub-nanopore that is sputtered through a nanometer-thick membrane with a tightly focused high-energy electron beam, is designed to be about the size of an amino acid (AA), which accounts for the extreme sensitivity. When the membrane is immersed in electrolyte and a voltage is applied across it, the electric force on the ions in solution produces a current through the sub-nanopore. Subsequently, when a charged denatured protein is impelled by the same electric force through the sub-nanopore, the open pore ionic flow is blocked by the AAs in the pore waist. The resulting current blockade signals modulated by the AA sequence constituting the protein are called a nanospectrum. A nanospectrum consists of a list of data points, each represented by a time and the current blockade signal measured at the time. It has been shown that the nanospectrum is correlated with the volume of AAs occluding the pore, so the AA sequence constituting the protein can be read from the fluctuations in the nanospectrum^1,3^.

Currently, mass spectrometry (MS) is the leading technology for protein identification^6^. Whereas bottom-up MS analyzes short peptides resulting from proteolytic digestion, top-down MS is capable of analyzing intact proteins^7^. However, MS-based protein identification has fundamental limitations in sensitivity and measurable molecular masses^8^. MS detection requires between an attomole and femtomole of protein, making it challenging to identify low abundance peptides or proteins. Moreover, MS often fails to achieve high sequence coverage for long proteins. Bottom-up MS identifies some peptides of long proteins but does not offer high sequence coverage. Top-down MS provides whole sequence coverage of proteins, but the measurable mass of a protein is limited due to mass spectrometers’ capacity.

Sequencing proteins with a sub-nanopore could be a disruptive technology for several reasons^2^. First, a sub-nanopore reads single protein molecules, significantly increasing the dynamic range of protein identification. Because of this, single-molecule protein sequencing has many applications in low abundance protein analysis and single-cell proteomics. Second, sub-nanopore sequencing is not limited by the molecular weight of the protein. In principle, a sub-nanopore could read thousands of AAs in a single molecule. Third, a sub-nanopore is capable of analyzing the prevalence of heterogeneity in mRNA translation^9^ and post-translational modifications (PTMs)^10^ by *direct* protein-level analysis. So far, the analysis of blockade currents in nanometer-diameter pores has been applied successfully to call the sequence of bases constituting DNA and RNA. Many methods have been developed to improve nanopore DNA reads' base calling accuracy, including hidden Markov and neural network models^11^. As a result, the base-calling accuracy has been improved from 63% to > 95% within the last several years^12–14^. Similarly, computational methods can potentially improve the accuracy of sub-nanopore protein sequencing. However, detecting the AA sequence in a protein with a sub-nanopore is more exacting than discriminating the four bases that constitute DNA with a nanopore, and efficient tools are lacking for the analysis of the nanospectrum produced when a protein translocates across a membrane through a sub-nanopore.

The interpretation of sub-nanopore nanospectra still presents a daunting challenge for detection and identification. Current blockade signals are determined mainly by the volume of the AA in the pore. Moreover, there are large variances in the measured blockades that may be due to other factors, which include electrical and molecular configurational noise, the AA mobility and hydrophobicity in the pore, and the neighboring AAs in the sequence. Even if it is detected, calling the AA is confounded by the protein's primary structure, which is drawn from twenty proteinogenic AAs. Beyond just the twenty proteinogenic AAs, the challenge confronting direct protein sequencing is compounded by protein isoforms derived from closely related duplicate genes or the same gene by alternative splicing, proteolytic cleavage, somatic recombination, or PTMs^10,15^. In a groundbreaking effort, Kolmogorov et al. first tackled the analysis by benchmarking several machine learning models and presented an alignment algorithm for protein identification using only a few nanospectra^16^, but due to the noise, it remained problematic to identify a protein by searching nanospectra against a protein database of the size of the human proteome.

The sub-nanopore technology has advanced rapidly in the past several years, and it is now capable of measuring the volumes of single AAs instead of several consecutive AAs. So, with the proper computational tools, it should be possible to decode single AAs directly using nanospectra. Here, several computational methods are presented for processing nanospectra and predicting theoretical nanospectra from protein sequences. These methods promise to improve the accuracy of theoretical nanospectral prediction and increase the Pearson correlation coefficient (PCC) between the empirical and theoretical nanospectra to > 0.9.

## Methods

### Peptide synthesis

Two carrier-free peptides were used in experiments (Anaspec, Fremont, CA): amyloid beta 42 (Aβ_1–42_) (DAEFRHDSGYEVHHQKLVFFAEDVGSNKGAIIGLMVGGVVIA), and a scrambled variant with the same chemical constituency as amyloid beta 42 (SAβ_1–42_) (AIAEGDSHVLKEGAYMEIFDVQGHVFGGKIFRVVDLGSHNVA). The two peptides were reconstituted according to the protocols offered by the manufacturer (Anaspec, Fremont, CA). Typically, the peptides were reconstituted at high (100 µg/ml) concentration in phosphate-buffered saline (1 × PBS) without adding bovine serum albumin (BSA) to avoid false readings. From this solution, aliquots diluted to 2 × the concentration of denaturant with 50 pM protein, 20–100 µM beta-mercaptoethanol (BME), 250 mM *NaCl* with 2–5 × 10^–3^% sodium dodecyl sulfate (SDS) were vortexed and heated to 85 °C for 120 min. The solution was allowed to cool (to 5 °C) and added in 1:1 proportion with the (75 µL) electrolyte in the reservoir of the polydimethylsiloxane (PDMS) microfluidic device bound to the silicon chip supporting the membrane with a pore through it housed in a 5 °C cold room^4^.

### Sub-nanopore fabrication and visualization

Briefly, a custom-made 5 nm thick amorphous silicon (a-*Si*) membrane (SiMPore, Inc. West Henrietta, NY) was sputter-deposited on a 50 nm thick thermal *SiO*_2_ layer grown on a float-zone silicon handle 100 µm thick and subsequently capped with another *SiO*_2_ layer with the same 25–50 nm thickness followed by the deposition of 150 nm of tetraethyl orthosilicate (TEOS). A membrane < 4–5 μm on-edge was revealed by an ethylene diamine and pyrocatechol chemical etch of the silicon through a silicon nitride window defined by photolithography on the polished back-side of the handle wafer. Finally, a buffered oxide etch (10:1 BOE) was used to remove the oxide to produce an a-*Si* membrane, which ranged from 3.5 to 6 nm thick.

Just prior to loading it into the transmission electron microscopy (TEM) column, the membranes were plasma cleaned using Tergeo-EM (PIE Scientific, Union City, CA, USA). The Tergeo-EM was operated at 10 W using an 80% Ar + 20% O_2_ gas feed in a down-stream, pulse mode (1/16 duty-cycle, which was cycled twice for a total exposure of 2 min) such that the samples were actually outside the plasma (to eliminate sputtering) and subjected to only extremely short plasma pulses (to reduce the intensity). Subsequently, a pore was sputtered through the thin a-*Si* membrane using a tightly focused, high-energy (300 kV) electron beam carrying a current ranging from 300 to 800 pA (post-alignment) in a Scanning Transmission Electron Microscope (STEM, FEI Titan 80–300 or FEI Themis Z, Hillsboro, OR) with a Field Emission Gun (FEG).

After sputtering, the pore was re-acquired with either High-Resolution Transmission Electron Microscopy (HRTEM) or High-Angle Annular Dark Field (HAADF-)STEM. To minimize beam damage, the pores were examined using low beam current (< 10–30 pA) or low energy (80 kV) or both. The illumination convergence angle in the Titan was typically α = 10 mrad at 300 kV, whereas in the Themis Z, α = 18 mrad at 300 kV or α = 27.1 mrad at 80 kV with a monochromator limiting the energy dispersion in the range 200–220 mV at 80 kV according to Electron Energy Loss Spectroscopy (EELS).

### Microfluidics

The microfluidic device similar to that used in these measurements was described in an earlier report^5^. Tersely, the silicon chip supporting the membrane with a single pore through it with or without a polyimide laminate was bonded to a polydimethylsiloxane (PDMS, Sylgard 184, Dow Corning) microfluidic device formed using a mold-casting technique. The microfluidic device consisted of two microchannels (each 250 × 75 μm^2^ in cross-section) connected by a via that could be as small as 25 μm in diameter. A tight seal was formed between the silicon chip containing the a-*Si* membrane with the pore in it and the PDMS *trans*-side of the microfluidic channel with a plasma-bonding process (PDS-001, Harrick Plasma, Ithaca, NY). Subsequently, two separate *Ag/AgCl* electrodes (Warner Instruments, Hamden, CT) were embedded in each channel to independently and electrically address the *cis-* and *trans*-sides of the membrane. Likewise, the two microfluidic channels were also connected to external pressure and fluid reservoirs through polyethylene tubing at the input and output ports. The port on the *cis*-side was used to convey proteins to the pore.

### Low-noise electrical measurements

The electrical measurements followed a procedure similar to that described elsewhere^5^. To perform blockade current measurements, first, the sub-nanopore was wetted by immersion in de-gassed 250 mM *NaCl* electrolyte for 1–3 days. Subsequently, a transmembrane voltage bias (< 700 mV) was applied to the reservoir (containing 75 µL of electrolytic solution and 75 μL of 2 × concentrated solution of protein and denaturant) relative to the ground in the channel using *Ag/AgCl* electrodes and the corresponding pore current was measured at 5 ± 0.1 °C using either an Axopatch 700B or an Axopatch 200B amplifier with an open bandwidth. The actual bandwidth was inferred from the rise-time to a sharp (10 ps rise-time) input pulse to be about 75–100 kHz, depending on the amplifier and the feedback. The analog data were digitized by a 16-bit DigiData 1550B data acquisition system (DAQ, Molecular Devices, Sunnyvale, CA) at a sampling rate of 500 kS/s and recorded in 3 min-long acquisition windows and stored in Axon binary files (ABFs). Generally, no blockades were observed beyond the noise in controls that comprised the electrolyte and the denaturants (SDS and BME), which were heated to 85 °C and then cooled without protein. A total of 12 ABFs were collected for Aβ_1–42_ and 70 ABF files for SAβ_1–42_.

### Data pre-processing

**Figure 1:**
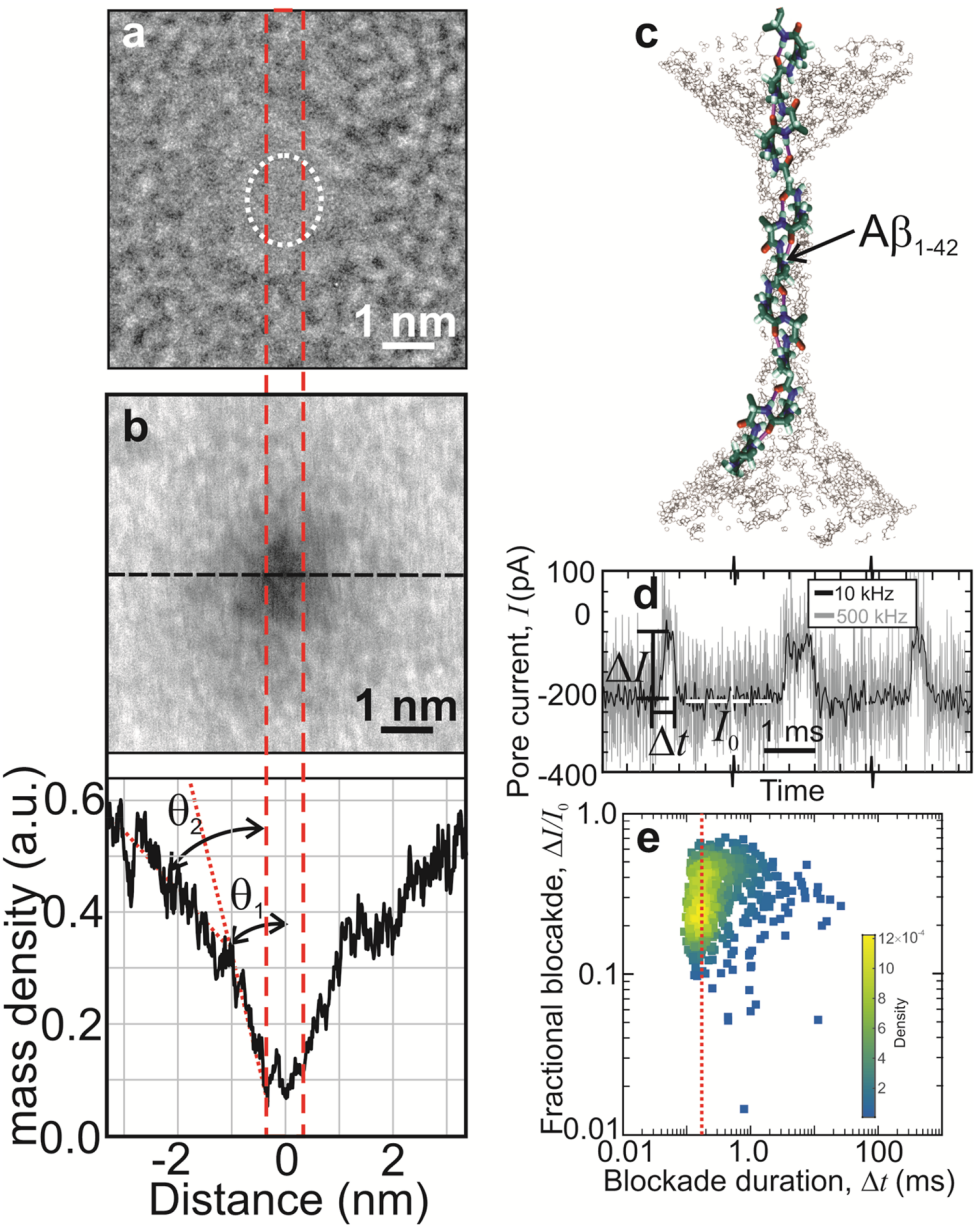
Improved read resolution and fidelity using a sub-nanopore through a thin laminated a-*Si* membrane. (**a**) A TEM image is shown *in vacuo* of a nanopore immediately after sputtering through a nominally 5 nm thick a-*Si* membrane. The cross-section of the pristine pore was estimated from the shot noise associated with electron transmission through the pore to be about 1.0 × 1.5 nm^2^ (dotted circle). (**b**, top) An HAADF-STEM image, acquired with an aberration-corrected microscope is shown of the pore in (**a**) after exposure to the ambient. (**b**, bottom) The profile of the mass-density under the probe beam is shown taken along the dashed (horizontal) line in (**d**, top). The cross-section shrunk to about 0.65 nm × 0.87 nm, indicative of the growth of a native oxide in the pore waist. (**c**) A schematic representation is shown depicting a translocation of Aβ_1–42_ impelled by an electric force through a sub-nanopore. The actual pore is ghosted in the figure; only the peptide is represented. (**d**) Current traces (negative raw current) are shown that illustrate the distribution of the duration of the blockade currents associated with translocations of single molecules of Aβ_1–42_ through a sub-nanopore spanning an a-*Si* membrane at 0.6 V. The pore current was amplified over a > 75 kHz bandwidth and sampled at 500 kHz (gray line) to detect each residue in the peptide in a ∆*t* = 170 μs blockade. Another version of the same data, filtered with a 10 kHz eight-pole Bessel filter (black line), is also shown. The definition of the blockade current, ∆*I*, the blockade duration, ∆*t*, and the open pore current, *I*_0_, are indicated. Higher current (negative raw current) values correspond to larger blockade currents. (**e**) A heat map is shown that illustrates the distribution of fractional blockades relative to the open pore current (Δ*I/I*_0_) versus the blockade duration (∆*t*) associated with single denatured Aβ_1–42_ molecules translocating through a sub-nanopore acquired at 0.6 V. The red dotted line shows the position of 170 μs.

The nanospectra in ABFs were extracted using a homemade MATLAB software package based on OpenNanopore (version 1.2)^17^. Nanospectra with a relatively long duration provided useful information for AA sequencing, but those that are too short did not. So, the nanospectra with a duration shorter than 170 µs were ignored. The duration threshold (170 μs) was determined based on the heatmap of the durations of nanospectra (Fig. 1e), which shows that the durations have the highest frequency at 170 μs and that the median of the durations is about 170 μs. Based on the observation, we kept only about half of all the nanospectra whose durations were longer than 170 μs.

The duration for a peptide in the sub-nanopore ranged from tens of microseconds to tens of milliseconds, and the numbers of data points in nanospectra vary dramatically. To address the variation in blockade duration, it was assumed that each nanospectrum represented the same pattern of fluctuations and so it was downsampled into a resampled nanospectrum of 500 data points by averaging neighboring data points when the nanospectrum contains > 500 data points and oversampled into a resampled nanospectrum of the same size by interpolating between neighboring data points when the nanospectrum contains < 500 data points. The nanospectra after resampling had the same number of data points and the same average number of data points for an AA. Thus, a consensus formed from these nanospectra represents signals irregularly (nonuniformly) sampled. Regardless of the duration, consensuses formed this way can inform on each AA in the sequence^3,18–22^.

### Features for AAs

Linear regression was employed to predict the current blockade signals (theoretical nanospectra) of AAs in peptides, which were represented using several encoding methods. A given peptide sequence ${a}_{1},{a}_{2},\dots ,{a}_{n}$ was converted to a list of AA volumes: ${b}_{0},{b}_{1},\dots , {b}_{n+1}$, where ${b}_{0}={b}_{n+1}=0$ and ${b}_{i}$ is the AA volume^23^ corresponding to ${a}_{i}$ for $1\le i\le n$. The first encoding method is based on single AA volumes: an AA ${a}_{i}$ is represented by its volume ${b}_{i}$. The second encoding method is based on the volumes of the AA and its two neighboring ones: an AA ${a}_{i}$ is represented by two values ${b}_{i}$ and ${b}_{i-1}+{b}_{i+1}$. In the third encoding method, the 20 AAs are divided into 4 groups based on their volumes: minuscule (G, A, S, C), small (T, D, P, N, V), intermediate (E, Q, H, L, I, M, K), and large (R, F, Y, W)^16^. So, given a peptide ${a}_{1},{a}_{2},\dots ,{a}_{n}$, let ${M}_{i}=1$ if ${a}_{i}$ is a minuscule AA and ${M}_{i}=0$ otherwise, for $1\le i\le n$. Specifically, ${M}_{0}={M}_{n+1}=0$. For position *i* in the peptide, we extract four features based on the volume of ${a}_{i}$. The first feature ${x}_{M}$ is the volume of the AA if it is a minuscule one, and 0 otherwise, defined as ${x}_{M}={M}_{i}{b}_{i}$. The features for small (${x}_{S}$), intermediate (${x}_{I}$), and large (${x}_{L}$) AAs are defined similarly. The three encoding methods are referred to as single AA volume (1AAV), three AA volume (3AAV), and AA group (AAG) methods, respectively.

The three encoding methods were further extended to include a position feature, which represents the distance between the AA and the N- or C-terminus. When the distance is larger than 4, the AA is treated as a middle one and the feature is set to 5. For ${a}_{i}$ with position *i*, the position feature ${x}_{P}$ is:

$$x_{P} = \left\{ {\begin{array}{*{20}l} i \hfill & {{\text{if}}\,\,i \le 4,} \hfill \\ 5 \hfill & {{\text{if}}\,\,i > 4\,\,\,{\text{and}}\,\,\,i < n - 3,} \hfill \\ {n - i + 1} \hfill & {{\text{if}}\,\,i \ge n - 3.} \hfill \\ \end{array} } \right.$$

The three encoding methods with the position feature are referred to as 1AAV-P, 3AAV-P, and AAG-P, respectively.

### Orientation of the nanospectra

It was assumed that a nanospectrum of a peptide had two possible orientations: a *forward* nanospectrum enters the pore axis N-terminus first and a *backward* nanospectrum C-terminus first. Let $S={s}_{1}{s}_{2}\dots {s}_{m}$ be an empirical nanospectrum with *m* data points, where ${s}_{i}$ is the current blockade signal at time point *i*, and ${S}^{^{\prime}}={s}_{m}{s}_{m-1}\dots {s}_{1}$ the flipped nanospectrum of *S*. To account for the two orientations, a theoretical nanospectrum $T={t}_{1}{t}_{2}\dots {t}_{m}$ of the peptide derived from the 1AAV model and linear interpolation was generated and compared with empirical nanospectra. $PCC(S,T)$ represents the PCC of an empirical nanospectrum *S* and the corresponding theoretical nanospectrum *T*. If $\mathrm{PCC}\left(S,T\right)>\mathrm{PCC}({S}^{^{\prime}},T)$, then *S* is forward, otherwise, backward. The backward nanospectra were flipped so that all nanospectra had the same orientation.

### Dynamic time warping

Let $S={s}_{1}{s}_{2}\dots {s}_{m}$ and $T={t}_{1}{t}_{2}\dots {t}_{m}$ be an empirical and a theoretical nanospectra of a peptide, respectively. Both *S* and *T* were normalized to have zero mean and unit variance. Let $S[i,j]$ represent the subsequence ${s}_{i}{s}_{i+1}\dots {s}_{j}$ of *S*. Because the velocity of the AAs moving through the sub-nanopore might vary, ${s}_{i}$ and ${t}_{i}$ might correspond to different AAs in the peptide. To address the problem, dynamic time warping (DTW)^24^ was used to adjust the time-axis of the data points in *T* to match the empirical data points in *S* (Fig. S1). DTW tends to have the singularity problem by matching the signal of a short time window to that of a long time window^25^, so a constraint was introduced such that the ratio between any two time periods matched by DTW should be between 2/3 and 3/2. That is to say, 6 data points in *T* can be matched with at least 4 data points and at most 9 data points in *S*. The squared error was used to measure the distance between two data points ${s}_{i}$ and ${t}_{i}$, i.e.,$d\left({s}_{i},{t}_{i}\right)={({s}_{i}-{t}_{i})}^{2}.$

We fill out a 2-dimensional $(m+1)\times (m+1)$ table *D*, in which $D[i,j]$ stores the minimum distance between $S[1,i]$ and $T[1,j]$ after time warping. The recurrence function for computing $D[i,j]$ is shown Step 4 in Fig. S1. Because at least 2 data points in *S* are needed to match 3 data points in *T* and vice versa, the singularity problem is solved. The algorithm's time complexity is $O({m}^{2})$ .

### Consensus nanospectra

To reduce the noise in nanospectra, a consensus spectrum of a peptide was formed by combining all nanospectra of the peptide. Accordingly, if ${S}_{1}, {S}_{2}, \dots , {S}_{n}$ are the nanospectra of a peptide after orientation correction and ${S}_{i}[j]$ is the current blockade signal for the *j*th point in *S*_*i*_ for $1\le i\le n$ and $1\le j\le m$, then the consensus nanospectrum *S* was formed by taking the average current blockade signals of the nanospectra. That is to say, the consensus signal $S\left[j\right]=\frac{{\sum }_{i=1}^{n}{S}_{i}[j]}{n}$ for $1\le j\le m$ . The nanospectrum *S* is called the *average consensus nanospectrum* of the peptide.

One limitation of the average consensus approach was that it failed to consider the variance in the velocity with which AAs pass the sub-nanopore. The relative dwell time of an AA in a peptide molecule is the ratio between the AA dwell times and the whole molecule. The relative dwell times in nanospectra for the same AA in the peptide could be different. Owing to this variance, the current blockade signals ${S}_{1}\left[j\right], {S}_{2}\left[j\right],\dots , {S}_{n}[j]$ for the same position *j* could originate from different AAs and so the average current blockade signal may be an inaccurate consensus of the nanospectra.

Similar to multiple sequence alignment^26^, a progressive method was used to improve the quality of average consensus nanospectra with high-quality empirical nanospectra (Fig. S2). According to this algorithm, DTW was used to align each empirical nanospectrum with the average consensus nanospectrum, and then each was ranked in the increasing order of the distance. The top *t* empirical nanospectra (*t* = 50 in this analysis) were chosen to update the consensus. The best empirical nanospectrum was first aligned with the average consensus nanospectrum, and the average consensus nanospectrum was then updated by forming a weighted average with the best empirical nanospectrum. This step was repeated for the top *t* nanospectra. Specifically, to update the consensus using the *i*th empirical spectrum, the weight for the consensus was *u* + *i*-1 and that for the highly ranked empirical spectrum was 1, where *u* is the weight for the original consensus (*u* = 30 in the experiments). The updated consensus nanospectrum is referred to as *the alignment consensus nanospectrum* of the peptide. Notably, it is possible that AAs back-step (backward fluctuations) when they pass the sub-nanopore. The back-stepping identification problem is challenging and has not been solved due to noise signals in nanospectra, and the DTW method cannot identify AA back-stepping events.

The functions for nanospectral analysis were coded in Python. All the data processing was performed on a computer with an Intel Core i7-6700 3.4 GHz CPU and 16 GB memory.

## Results

### Sub-nanopore fabrication and characterization

A sub-nanopore sputtered through a thin, nominally 5 nm thick, a-*Si* membrane was used to analyze the peptides. The thickness was important because it affected the field distribution in the pore and therefore the resolution of a read. A pore was sputtered in the window through the a-*Si* membrane using a tightly focused, high energy (300 keV) electron beam formed in either a STEM Titan or Themis Z STEM (“Methods” and Supplementary Information S1). Subsequently, the pore was visualized in situ with HRTEM immediately after sputtering to reveal a 1.0 × 1.5 nm^2^-cross-section at the waist defined by the shot noise (Fig. 1a). However, the pore topography was likely affected, not only by electron-beam sputtering but also by oxidation in the ambient. This is likely because after exposure to the ambient for 1–3 days, the same pore was re-acquired and the topography visualized with HAADF using an aberration-corrected (Themis Z) STEM (Fig. 1b) to reveal a smaller lumen. Based on images like this, the pore topography was bi-conical with a steep cone angle > 7.4° that broadened to 16° near the orifice with an irregular waist 0.65 nm × 0.87 nm in cross-section.

If the bi-conical topography focused the electric field to a sub-nanometer extent near the waist then it followed that a blockade mainly measured the occluding volume due to the AAs in the waist (Fig. 1c). So, if only a few AAs occupied the waist at a time, it was reasoned that the blockade current would mainly measure the volume associated with those AAs. Likewise, it has been shown empirically that the small size of a sub-nanopore knocks-down the mobility of de-hydrated ions^5^, so it should also affect the AA mobility in the same way. Doubtless other AAs outside the waist would still contribute at least marginally to the blockade current and the mobility in the pore.

### Measurements of the blockade current

Measurements of fluctuations in the blockade current through a sub-nanopore were used to analyze the AA sequence of two synthesized peptides: a 42-residue (human) amyloid-β (Aβ)-protein fragment Aβ_1–42_ and a scrambled variant SAβ_1–42_ of it (“Methods”). The blockade is defined as the difference between the open sub-nanopore current ${I}_{0}$ and the current *I* in the peptide translocation, that is, $\Delta I={I}_{0}-I$ . When a nearly pH-neutral (pH 6.6 ± 0.1) solution containing denatured Aβ_1–42_ or SAβ_1–42_ peptides was introduced on the *cis*-side of a sub-nanopore with a voltage of 0.40–0.6 V applied across the membrane, blockades were observed almost immediately (Fig. 1d). The blockades were attributed to the translocation of rod-like single peptides across the membrane through the sub-nanopore (Fig. 1c). To account for the rapidity of the translocation, the electrical signal was amplified over a 50–75 kHz bandwidth and sampled at 500 kS/s. Accordingly, the signal was obscured by electrical noise.

Clusters of blockades were selected in a range demarcated by the Nyquist sampling rate corresponding to at least 0.5 samples per AA (with a blockade duration ∆*t* > 42 μs for Aβ_1–42_ and SAβ_1–42_ amplified with a 75–100 kHz bandwidth, and then sampled at 500 kS/s). To facilitate comparisons, the selected blockades of Aβ_1–42_ were classified by the duration of the blockade (∆*t*) and the fractional blockade, which is the ratio between in the blockade current and the open sub-nanopore current (∆*I/I*_0_). The aggregate data was binned (150 × 150), smoothed, and then represented by normalized heat maps of the probability density functions (PDFs) reflecting the number and distribution of blockades (Fig. 1e).

### Preprocessing of nanospectra

**Figure 2:**
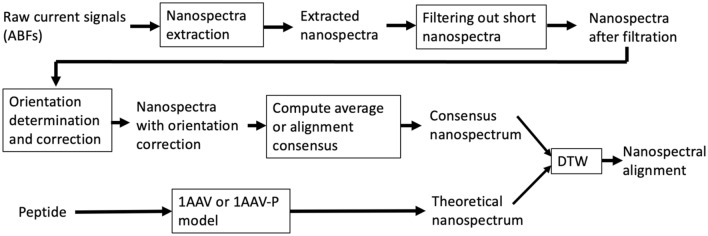
Overall scheme of nanospectral data analysis. Raw current blockade signals stored in ABFs are analyzed by a homemade software package based on OpenNanopore to extract nanospectra, which are filtered to remove those with short duration times. The orientations of nanospectra are determined by comparing original and flipped empirical nanospectra with the theoretical nanospectra of the peptide, and backward empirical nanospectra are flipped. The averaging or progressive alignment method is employed to generate a consensus empirical nanospectrum. A theoretical nanospectrum is generated using the 1AAV or 1AAV-P model. Finally, the empirical consensus nanospectrum is aligned with the theoretical nanospectrum using DTW to find an alignment between the two nanospectra, and the PCC of the aligned nanospectra is computed.

The overall scheme of nanospectral data analysis is shown in Fig. 2. Nanospectra were first extracted from ABFs using a homemade software package (“Methods”) and then filtered based on their duration times. Almost all the raw nanospectra have a duration longer than 42 μs, whereas about half had a duration > 170 μs (Fig. 1e). Raw nanospectra that were too short in duration could not realistically inform on all the AAs with the limited bandwidth of the amplifier and the 500 kS/s sampling rate. On the other hand, raw nanospectra that were too long would likely muddle the interpretation of the signal because of (slip-stick) translocation kinetics^3^. In data preprocessing, raw nanospectra with a long duration were still included because they can provide some information of AAs, and all raw nanospectra with a duration < 170 μs were removed, resulting in 475 and 2000 nanospectra for Aβ_1–42_ and SAβ_1–42_, respectively (“Methods”).

### Consensus nanospectra

The orientations of nanospectra were determined using the PCCs between empirical nanospectra and theoretical ones generated from the 1AAV model (“Methods”). Of the 475 Aβ_1–42_ nanospectra, the orientations of 268 were forward and 207 were backward. Of the 2000 SAβ_1–42_ nanospectra, 950 were forward and 1,050 were backward. Many empirical spectra have a slight difference between the PCCs of the original nanospectrum and the flipped one, making it challenging to determine their orientations confidently (Fig. S3).

**Figure 3:**
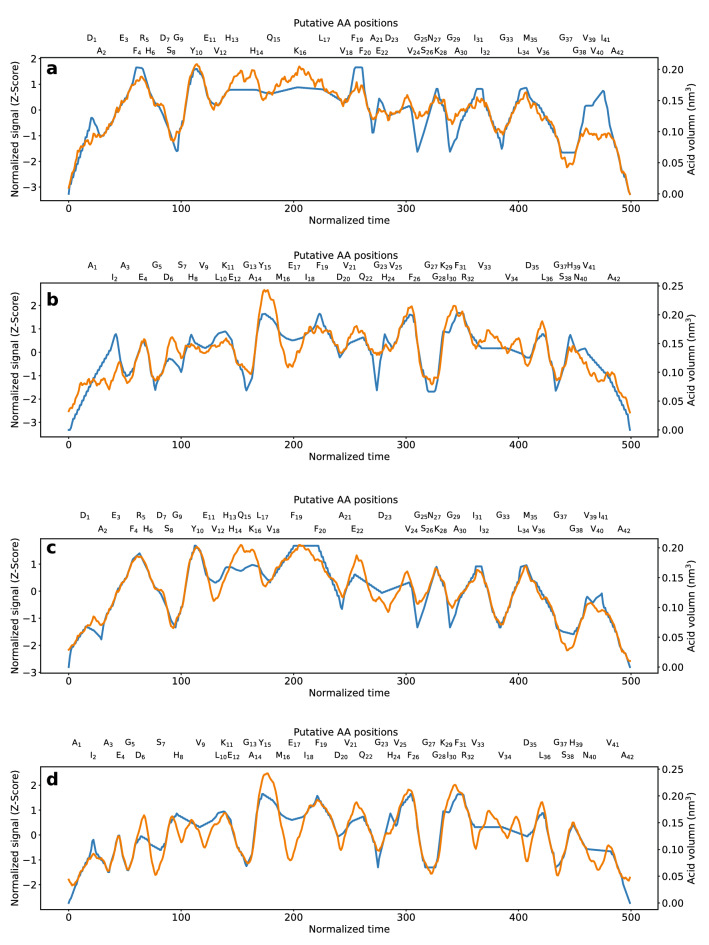
(**a**) A plot of a 475-blockade average consensus nanospectrum acquired at 0.6 V by forcing denatured Aβ_1–42_ through a sub-nanopore is shown versus normalized duration (orange line). Aligned with the empirical data is the corresponding 1AAV model (blue line) using DTW. The blockade current is correlated (PCC = 0.896) with the corresponding volume model. (**b**) A plot of a 2000-blockade average consensus nanospectrum acquired at 0.6 V by forcing denatured SAβ_1–42_ through a sub-nanopore is shown versus normalized duration (orange line). Aligned with the empirical data is the corresponding 1AAV mode (blue line) with DTW. The empirical consensus is correlated (PCC = 0.880) with the corresponding 1AAV model. (**c**) The alignment consensus nanospectrum (orange line) of Aβ_1–42_ is aligned with the 1AAV-P model (blue line) with PCC = 0.954. (**d**) The alignment consensus nanospectrum (orange line) of SAβ_1–42_ is aligned with the 1AAV-P model (blue line) with PCC = 0.903.

An average consensus nanospectrum of the peptide was formed to recover reproducible fluctuations in the blockade signal from irreproducible noise. The average consensus nanospectra were aligned with the corresponding theoretical nanospectra (1AAV) using DTW (“Methods”). It was compelling that the amplitude fluctuations in the average consensus nanospectra (Fig. 3a,b; orange lines) were highly correlated to the theoretical nanospectra (Fig. 3a,b; blue lines). Strikingly, the amplitude of the fluctuations tracked the AA volumes ascribed to the primary structure of Aβ_1–42_ with PCC = 0.896 (Fig. 3a). A sub-nanopore assay of SAβ_1–42_, consisting of a different sequence of the same AAs produced conspicuous differences in the fluctuation pattern (Fig. 3b), however, and was correlated (PCC = 0.880) to the corresponding 1AAV model for the scrambled sequence.

The average consensus nanospectra for Aβ_1–42_ and SAβ_1–42_ were further improved by using the progressive alignment method (“Methods”). The PCCs for the alignment consensus nanospectra and the theoretical nanospectra (1AAV) were 0.919 and 0.876 for Aβ_1–42_ and SAβ_1–42_, respectively (Fig. S4). The progressive alignment method increased the quality of the Aβ_1–42_ consensus nanospectrum but slightly lowered the quality of the SAβ_1–42_ consensus nanospectrum. It is likely that the top empirical nanospectra of Aβ_1–42_ forming the consensus might be of higher quality than those of SAβ_1–42_, so they could improve the consensus.

The correlations that developed between the consensus nanospectra and the corresponding volume models were important for two reasons. The fluctuations translated to reads with (nearly) single residue resolution, which could facilitate calling AAs as it alleviates the analytical and computational burden associated with ferreting out the identity of multiple monomers producing a fluctuation in a blockade. Second, it was also important because the fidelity proves that the signal–noise ratio can be improved with a reduction of the parasitic capacitance and with enough signal averaging, even with a high sampling frequency and no filtering. The correlation between the empirical consensuses and the corresponding volume models used for AA calls was still imperfect.

### Prediction of blockade currents

Seeking further refinement of the model, the alignment consensus and theoretical (1AAV) nanospectra of Aβ_1–42_ were normalized using zero mean and unit variance to form the Z-score, and then 42 data points were extracted from the alignment between the consensus and theoretical nanospectra. Each data point corresponded to an AA in the peptide. Likewise, 42 data points were extracted from the SAβ_1–42_ alignment consensus nanospectrum. Then linear regression was used to predict blockade signals with six encoding methods: 1AAV, 3AAV, AAG, 1AAV-P, 3AAV-P, and AAG-P (“Methods”). Prediction accuracy was evaluated using two-fold cross-validation: first, the training data were the Aβ_1–42_ data points and the validation data were the SAβ_1–14_ data points, and then the training and validation data sets were swapped. The error function was the mean squared error (MSE).

The methods with the AA position feature outperformed those without the feature, showing that the AA positions affect their current blockade signals, especially for those near the N- or C-terminus (Table 1). The AAs near the N- or C-terminus tended to have lower blockade signals than those in the middle. The 1AAV-P method obtained the lowest validation error. 3AAV-P and AAG-P reported better training errors than 1AAV-P, but their validation errors were worse than 1AAV-P, showing that they might have an overfitting problem due to the limited size of the training data. Using theoretical nanospectra predicted by the 1AAV-P model, the PCCs between theoretical and consensus nanospectra were further improved to 0.954 and 0.903 for Aβ_1–42_ and SAβ_1–42_, respectively (Fig. 3c,d).

**Table 1:** Comparison of six encoding methods for predicting blockade signals.

	1AAV	3AAV	AAG	1AAV-P	3AAV-P	AAG-P
Training error (MSE)	0.256	0.229	0.221	0.193	0.186	**0.169**
Validation error (MSE)	0.256	0.259	0.289	**0.211**	0.223	0.241

Significant values are in bold.

We also tested support vector machine (SVM) regression and random forest regression, but their performance was not as good as linear regression. The training data set with 42 data points was not enough for training complex machine learning models and may have caused an overfitting problem and lowered the prediction accuracy for the SVM and random forest regression models. Because the linear regression model is simpler than the other two, training it with the small data set did not result in a severe overfitting problem and provided better prediction accuracy than the other two.

### Statistical significance of nanospectral identifications

We generated 10,000 random peptides 42 AAs long and their corresponding theoretical nanospectra using the 1AAV-P method. Subsequently, DTW was used to align the theoretical spectra and the alignment consensus nanospectra of Aβ_1–42_ and SAβ_1–42_ separately. The average PCC between the random peptides and the consensus nanospectrum was 0.837 for Aβ_1–42_ and 0.803 for SAβ_1–42_ (Fig. S5). Based on the PCCs of the random peptides, the estimated *p*-value of the match between the theoretical and alignment consensus nanospectra was 0.0002 for Aβ_1–42_ and 0.0022 for SAβ_1–42_.

We also compared the alignment consensus nanospectrum of Aβ_1–42_ with the theoretical nanospectrum of SAβ_1–42_ using the 1AAV-P model. The PCC of the two nanospectra with DTW was 0.861 (Fig. S6a), which was consistent with the PCCs between the alignment consensus nanospectrum of Aβ_1–42_ and the theoretical nanospectra of random peptides (Fig. S5). Although DTW increases the PCC between an empirical nanospectrum and the theoretical nanospectrum of a random peptide, the PCCs of a random peptide and the peptide from which the experimental nanospectrum was generated can be separated. Similarly, the PCC (0.769) between the alignment consensus nanospectrum of SAβ_1–42_ and the theoretical nanospectrum of Aβ_1–42_ (Fig. S6b) was lower than that (0.903) of the alignment consensus and theoretical nanospectra of SAβ_1–42_.

## Discussion and conclusions

Various computational methods for signal processing, blockade current prediction, and identification of nanospectra using Aβ_1–42_ and SAβ_1–42_ peptides have been scrutinized for protein sequencing and identification. Since raw nanospectra are noisy, an indispensable pre-processing step is to use average nanospectra and alignment to obtain high-quality consensus nanospectra. Progressive alignment between the average consensus and top raw nanospectra could further improve the consensus of Aβ_1–42_, but not SAβ_1–42_. Apparently, the performance of the alignment method depends on the quality of raw nanospectra.

The experimental results showed that a sub-nanopore can identify purified peptides with only hundreds or thousands of molecules. Compared with MS, which needs hundreds of thousands of molecules for peptide identification, sub-nanopores increase the sensitivity in peptide identification by hundreds of folds. The high sensitivity of sub-nanopores will make it possible to identify and quantify low abundance proteins in single cells, which is still challenging for MS-based methods. In addition, sub-nanopores have inherent advantages in peptide/protein quantification. The abundance of a peptide/protein can be measured by counting the number of nanospectra reported in the data, which is much more accurate than MS-based peptide/protein quantification, which is hampered by the complexity in MS experiments, such as protein digestion and peptide ionization.

Six methods for predicting blockade signals of AAs were tested and benchmarked. Adding the positional information into blockade signal prediction improved the PCCs between theoretical and empirical nanospectra. Because only 84 data points were used for training and validation, only the 1AAV method showed similar accuracy in training and validation. The 3AAV and AAG methods obtained small prediction errors in the training data, but their validation errors were large. These methods have the potential to improve prediction accuracy, but more training data are needed to address the overfitting problem.

Many computational problems in nanospectral data analysis have not been well studied. For example, nanospectral clustering is an important pre-processing step for analyzing nanospectra of peptide mixtures, and predicting the peptide length of nanospectra is needed to identify truncated proteoforms. There are still no software tools for these problems. Accurate theoretical nanospectra can significantly increase the statistical significance of identifications in database search. So further improvement in the accuracy is needed for predicting theoretical nanospectra of peptides and those with PTMs—molecular dynamics simulations may be useful in this endeavor. De novo peptide sequencing from nanospectra is a challenging problem with high impact. A large nanospectral data set is also needed for training machine learning models and testing the performance of nanospectral data analysis methods.

## Supplementary Information

Supplementary Information.

## Data Availability

The nanospectral data are publicly available at https://massive.ucsd.edu/ (ID: MSV000089779).

## References

[1] Restrepo-Perez L, Joo C, Dekker C (2018). Paving the way to single-molecule protein sequencing. Nat. Nanotechnol..

[2] Timp W, Timp G (2020). Beyond mass spectrometry, the next step in proteomics. Sci. Adv..

[3] Dong Z, Kennedy E, Hokmabadi M, Timp G (2017). Discriminating residue substitutions in a single protein molecule using a sub-nanopore. ACS Nano.

[4] Kennedy E, Dong Z, Tennant C, Timp G (2016). Reading the primary structure of a protein with 0.07 nm(3) resolution using a subnanometre-diameter pore. Nat. Nanotechnol..

[5] Rigo E, Dong Z, Park JH, Kennedy E, Hokmabadi M, Almonte-Garcia L (2019). Measurements of the size and correlations between ions using an electrolytic point contact. Nat. Commun..

[6] Nilsson T, Mann M, Aebersold R, Yates JR, Bairoch A, Bergeron JJ (2010). Mass spectrometry in high-throughput proteomics: ready for the big time. Nat. Methods.

[7] Whitelegge J (2013). Intact protein mass spectrometry and top-down proteomics. Expert Rev. Proteomics.

[8] Angel TE, Aryal UK, Hengel SM, Baker ES, Kelly RT, Robinson EW (2012). Mass spectrometry-based proteomics: Existing capabilities and future directions. Chem. Soc. Rev..

[9] Boersma S, Khuperkar D, Verhagen BMP, Sonneveld S, Grimm JB, Lavis LD (2019). Multi-color single-molecule imaging uncovers extensive heterogeneity in mRNA decoding. Cell.

[10] Aebersold R, Agar JN, Amster IJ, Baker MS, Bertozzi CR, Boja ES (2018). How many human proteoforms are there?. Nat. Chem. Biol..

[11] Wick RR, Judd LM, Holt KE (2019). Performance of neural network basecalling tools for Oxford Nanopore sequencing. Genome Biol..

[12] Rang FJ, Kloosterman WP, de Ridder J (2018). From squiggle to basepair: Computational approaches for improving nanopore sequencing read accuracy. Genome Biol..

[13] Schreiber J, Karplus K (2015). Analysis of nanopore data using hidden Markov models. Bioinformatics.

[14] Silvestre-Ryan J, Holmes I (2021). Pair consensus decoding improves accuracy of neural network basecallers for nanopore sequencing. Genome Biol..

[15] Smith LM, Kelleher NL (2013). Proteoform: a single term describing protein complexity. Nat. Methods.

[16] Kolmogorov M, Kennedy E, Dong Z, Timp G, Pevzner PA (2017). Single-molecule protein identification by sub-nanopore sensors. PLoS Comput. Biol..

[17] Raillon C, Granjon P, Graf M, Steinbock LJ, Radenovic A (2012). Fast and automatic processing of multi-level events in nanopore translocation experiments. Nanoscale.

[18] Fay G, Kang S (2013). Average sampling of band-limited stochastic processes. Appl. Comput. Harmon. Anal..

[19] Long DG, Franz ROW (2016). Band-limited signal reconstruction from irregular samples with variable apertures. IEEE Trans. Geosci. Remote Sens..

[20] Behmard H, Faridani A (2002). Sampling of bandlimited functions on unions of shifted lattices. J. Fourier Anal. Appl..

[21] Wang D, Liu X, Wu X (2020). Wang Z (2020) Reconstruction of periodic band limited signals from non-uniform samples with sub-Nyquist sampling rate. Sensors (Basel)..

[22] Margolis E, Eldar YC (2008). Nonuniform sampling of periodic bandlimited signals. IEEE Trans. Signal Process..

[23] Perkins SJ (1986). Protein volumes and hydration effects: The calculations of partial specific volumes, neutron scattering matchpoints and 280-nm absorption coefficients for proteins and glycoproteins from amino acid sequences. Eur. J. Biochem..

[24] Berndt DJ, Clifford J (1994). Using dynamic time warping to find patterns in time series.

[25] Keogh, E. J., & Pazzani, M. J., editors. Derivative dynamic time warping. Proceedings of the 2001 SIAM international conference on data mining (SIAM, 2001).

[26] Thompson JD, Higgins DG, Gibson TJ (1994). CLUSTAL W: Improving the sensitivity of progressive multiple sequence alignment through sequence weighting, position-specific gap penalties and weight matrix choice. Nucleic Acids Res..

