# Supplementary material for: Calling the amino acid sequence of a protein/peptide from the nanospectrum produced by a sub-nanometer diameter pore
Source: Sci Rep. 2022 Oct 25;12:17853. doi: 10.1038/s41598-022-22305-x (PMC9596471; doi:10.1038/s41598-022-22305-x)
Supplement: Supplementary file 1 — Supplementary Information. [file 41598_2022_22305_MOESM1_ESM.pdf]

Calling the Amino Acid Sequence of a Protein/Peptide from the Nanospectrum Produced by a Sub-nanometer Diameter Pore (Supplementary Material)

Xiaowen Liu[1,2,*], Zhuxin Dong[3] and Gregory Timp[3,*]

1. Tulane Center for Biomedical Informatics and Genomics, Tulane University, New Orleans, LA, 70112, USA
2. Division of Biomedical Informatics and Genomics, Deming Department of Medicine, Tulane University, New Orleans, LA, 70112, USA
3. Departments of Electrical Engineering and Biological Sciences, University of Notre Dame, Notre Dame, IN, 46556, USA

* Corresponding authors

Input: An experimental nanospectrum $S = s_1 s_2 \ldots s_m$ and a theoretical nanospectrum $T = t_1 t_2 \ldots t_m$

Output: An alignment between S and T

1. Initialize an $(m+1) \times (m+1)$ table D by setting $D[0,0] = 0$, $D[1,1] = d(s_1, t_1)$ and other cells to ∞. We also assume $D[i,j] = \infty$ when $i < 0$ or $j < 0$.
2. **For** $i = 2$ to m **do**
3. **For** $j = 2$ to m **do**
4. $$D(i,j) = \min \begin{cases} D(i-2, j-2) + d(s_{i-1}, t_{j-1}) + d(s_i, t_j) \\ D(i-2, j-3) + d(S[i-1, i], T[j-2, j]) \\ D(i-3, j-2) + d(S[i-2, i], T[j-1, j]) \end{cases}$$
5. Use backtracking to find a best alignment between S and T.

SUPPLEMENTARY FIGURE 1. Dynamic time warping algorithm with a constraint. The distance between two data points $S[i-1, i]$ and three data points $T[j-2, j]$ is defined as $d(S[i-1, i], T[j-2, j]) = d(s_{i-1}, t_{j-2}) + d(s_i, t_j)$. The distance between $S[i-2, i]$ and $T[j-1, j]$ is defined as $d(S[i-2, i], T[j-1, j]) = d(s_{i-2}, t_{j-1}) + d(s_{i-1}, t_j) + d(s_i, t_j)$.

Input: An average consensus nanospectrum C, a list of experimental nanospectra $S_1, S_2, \ldots, S_n$ in the increasing order of their distances with C, and parameter u.

Output: An improved consensus nanospectrum

1. **For** $i = 1$ to m **do**
2. Use DTW to align C and S_i. The nanospectrum S_i after time warping is represented by S'_i.
4. Update C using the weighted average of C and S'_i. The weights of C and S'_i are $u + i - 1$ and 1, respectively.
4. Return C.

SUPPLEMENTARY FIGURE 2. Algorithm for improving the average consensus nanospectrum by alignment.

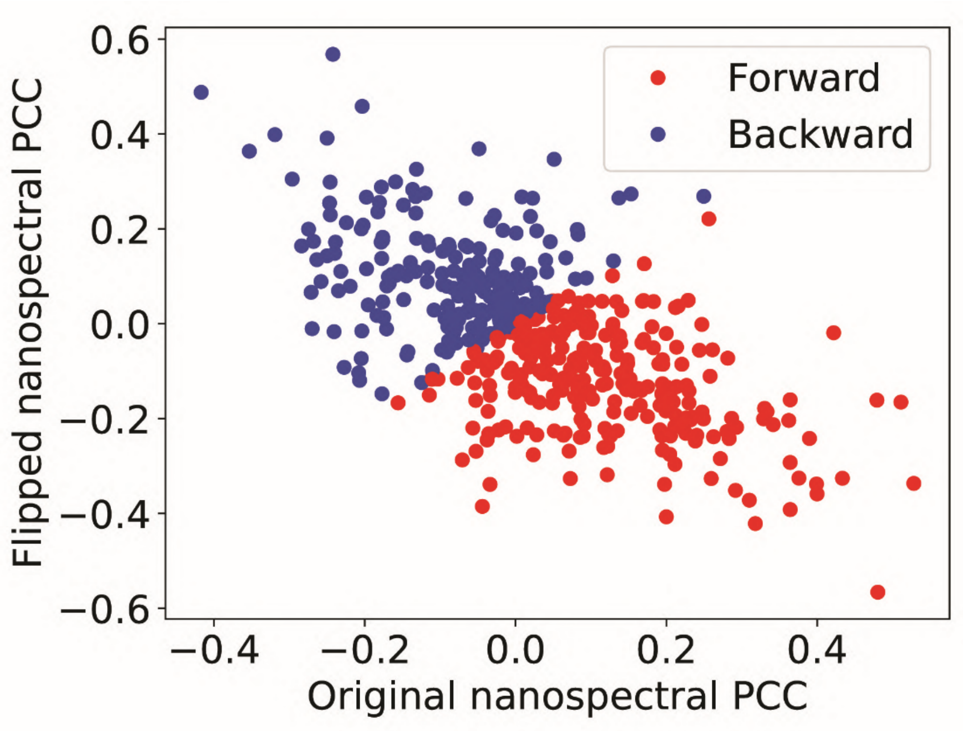

SUPPLEMENTARY FIGURE 3. **Forward and backward nanospectra**. PCCs of the empirical nanospectra and flipped empirical nanospectra of A$\beta_{1\text{-}42}$ compared with the theoretical nanospectrum generated using 1AAV model and linear interpolation.

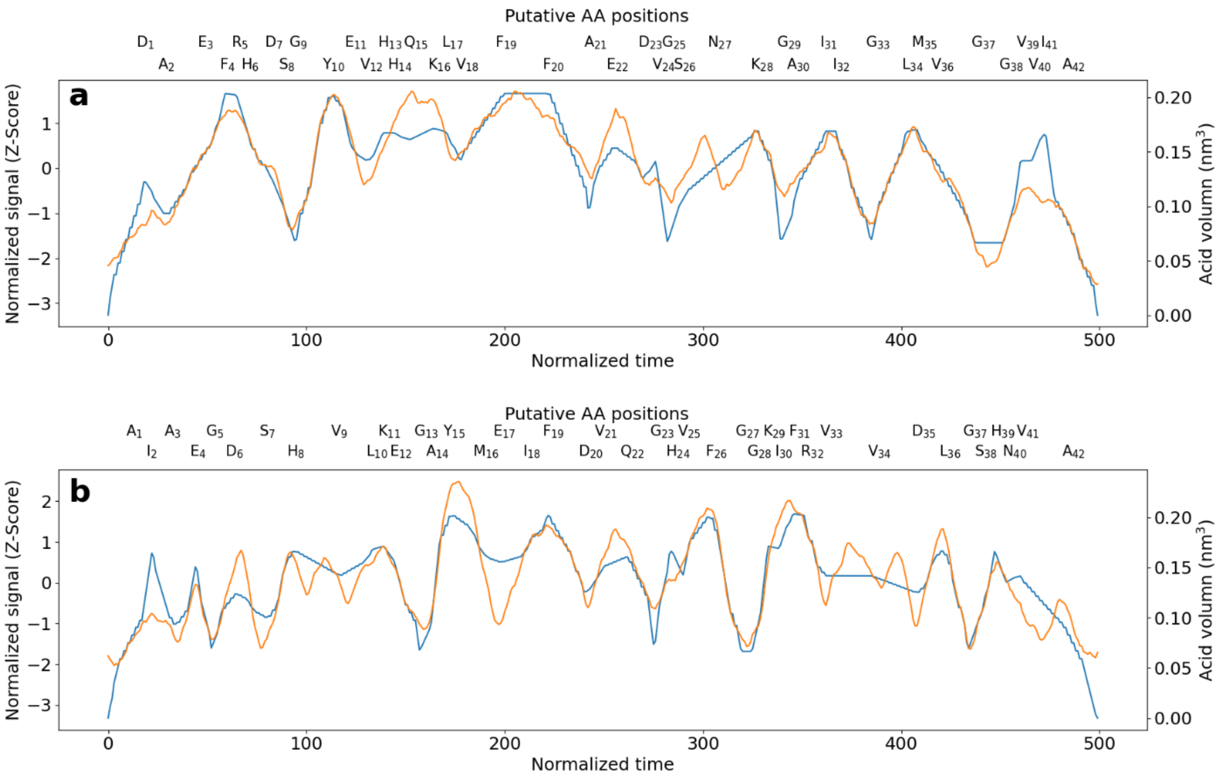

SUPPLEMENTARY FIGURE 4. Comparison of alignment consensus and theoretical nanospectra. (a) A plot of a 475-blockade alignment consensus nanospectrum of $A\beta_{1-42}$ is shown versus normalized duration (orange line). Aligned with the empirical data is the corresponding 1AAV model (blue line) using DTW. The alignment consensus was correlated (PCC = 0.919) with the corresponding volume model. (b) A plot of a 2000-blockade alignment consensus nanospectrum of $SA\beta_{1-42}$ is shown versus normalized duration (orange line). Aligned with the empirical data is the corresponding 1AAV mode (blue line) with DTW. The empirical alignment consensus was correlated (PCC = 0.876) with the corresponding 1AAV model.

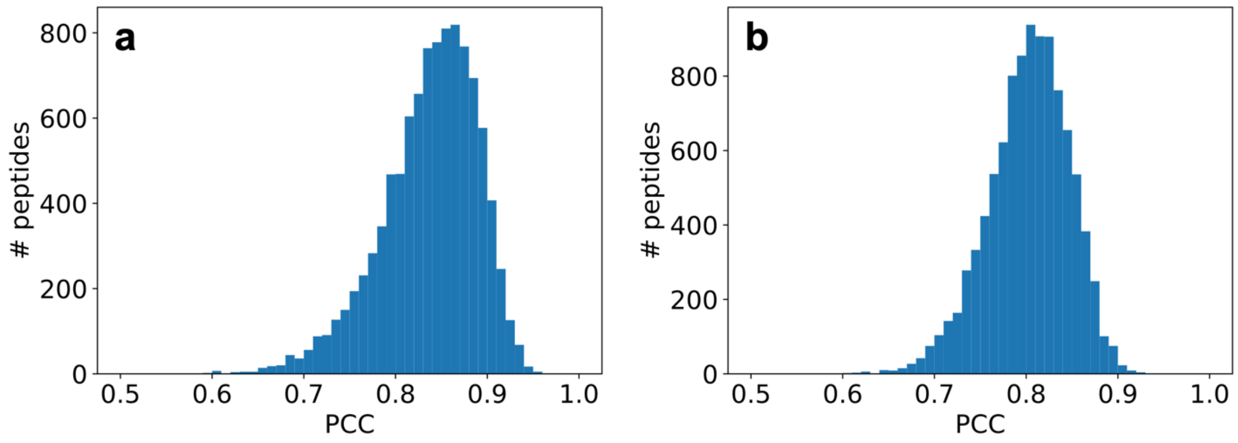

SUPPLEMENTARY FIGURE 5. Distribution of the PCCs between an alignment consensus nanospectra and the theoretical nanospectra of 10,000 random peptides after DTW. (a) PCCs of the alignment consensus nanospectrum of $A\beta_{1\text{-}42.}$ (b) PCCs of the alignment consensus nanospectrum of $SA\beta_{1\text{-}42.}$

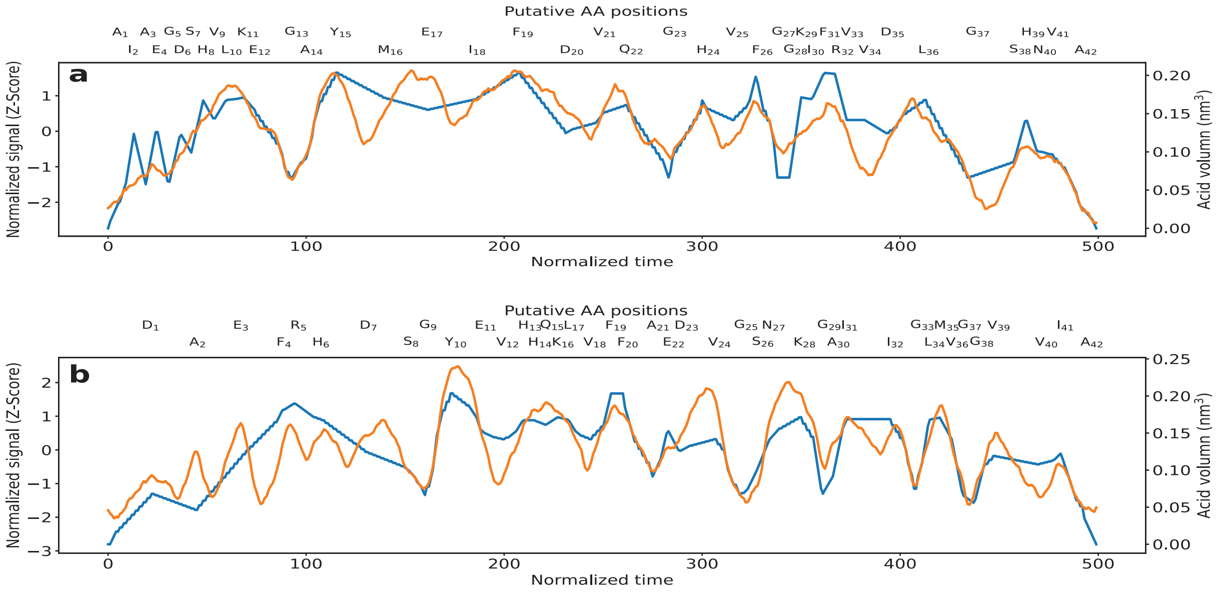

SUPPLEMENTARY FIGURE 6. Comparison of the alignment consensus nanospectrum of $A\beta_{1-42}$ ($SA\beta_{1-42}$) and the theoretical nanospectrum of $SA\beta_{1-42}$ ($A\beta_{1-42}$). (a) The 475-blockade alignment consensus nanospectrum of $A\beta_{1-42}$ (orange line) is aligned with the 1AAV-P model of $SA\beta_{1-42}$ (blue line) using DTW. The PCC between the alignment consensus and the 1AAV-P model is 0.861. (b) The 2000-blockade alignment consensus nanospectrum of $SA\beta_{1-42}$ (orange line) is aligned with the 1AAV-P mode of $A\beta_{1-42}$ (blue line) with DTW. The PCC between the alignment consensus of the 1AAV-P model is 0.769.

1. Raillon, C., et al., *Fast and automatic processing of multi-level events in nanopore translocation experiments.* Nanoscale, 2012. **4**(16): p. 4916-24.
2. Dong, Z., et al., *Discriminating residue substitutions in a single protein molecule using a sub-nanopore.* ACS Nano, 2017. **11**(6): p. 5440-5452.
3. Fay, G. and S. Kang, *Average sampling of band-limited stochastic processes.* Applied and Computational Harmonic Analysis, 2013. **35**: p. 527-534.
4. Long, D.G. and R.O.W. Franz, *Band-limited signal reconstruction from irregular samples with variable apertures.* IEEE Transactions on Geoscience and Remote Sensing, 2016. **54**(4): p. 2424-2436.
5. Behmard, H. and A. Faridani, *Sampling of bandlimited functions on unions of shifted lattices.* Journal of Fourier Analysis and Applications, 2002. **8**(1): p. 43-58.
6. Wang, D., et al., *Reconstruction of periodic band limited signals from non-uniform samples with sub-Nyquist sampling rate.* Sensors (Basel), 2020. **20**(21).
7. Margolis, E. and Y.C. Eldar, *Nonuniform sampling of periodic bandlimited signals.* IEEE Transactions on Signal Process, 2008. **56**(7): p. 2728-2745.
8. Perkins, S.J., *Protein volumes and hydration effects. The calculations of partial specific volumes, neutron scattering matchpoints and 280-nm absorption coefficients for proteins and glycoproteins from amino acid sequences.* European Journal of Biochemistry, 1986. **157**(1): p. 169-80.
9. Kolmogorov, M., et al., *Single-molecule protein identification by sub-nanopore sensors.* PLoS Computational Biology, 2017. **13**(5): p. e1005356.
10. Keogh, E.J. and M.J. Pazzani. *Derivative dynamic time warping*. in *Proceedings of the 2001 SIAM international conference on data mining*. 2001. SIAM.
